# Analysis of the regulation of fatty acid binding protein 7 expression in human renal carcinoma cell lines

**DOI:** 10.1186/1471-2199-12-31

**Published:** 2011-07-19

**Authors:** Naohisa Takaoka, Tatsuya Takayama, Takumi Teratani, Takayuki Sugiyama, Soichi Mugiya, Seiichiro Ozono

**Affiliations:** 1 Department of Urology, Hamamatsu University School of Medicine, Hamamatsu, Shizuoka, Japan; 2 Division of Development of Advanced Treatment, Jichi Medical University, Shimotsuke-shi, Japan

## Abstract

**Background:**

Improving the treatment of renal cell carcinoma (RCC) will depend on the development of better biomarkers for predicting disease progression and aiding the design of appropriate therapies. One such marker may be fatty acid binding protein 7 (FABP7), also known as B-FABP and BLBP, which is expressed normally in radial glial cells of the developing central nervous system and cells of the mammary gland. Melanomas, glioblastomas, and several types of carcinomas, including RCC, overexpress FABP7. The abundant expression of FABP7 in primary RCCs compared to certain RCC-derived cell lines may allow the definition of the molecular components of FABP7's regulatory system.

**Results:**

We determined *FABP7 *mRNA levels in six RCC cell lines. Two were highly expressed, whereas the other and the embryonic kidney cell line (HEK293) were weakly expressed *FABP7 *transcripts. Western blot analysis of the cell lines detected strong FABP7 expression only in one RCC cell line. Promoter activity in the RCC cell lines was 3- to 21-fold higher than that of HEK293. Deletion analysis demonstrated that three *FABP7 *promoter regions contributed to upregulated expression in RCC cell lines, but not in the HEK293 cell. Competition analysis of gel shifts indicated that OCT1, OCT6, and nuclear factor I (NFI) bound to the *FABP7 *promoter region. Supershift experiments indicated that BRN2 (POU3F2) and NFI bound to the *FABP7 *promoter region as well. There was an inverse correlation between *FABP7 *promoter activity and *BRN2 *mRNA expression. The FABP7-positive cell line's NFI-DNA complex migrated faster than in other cell lines. Levels of *NFIA *mRNA were higher in the HEK293 cell line than in any of the six RCC cell lines. In contrast, *NFIC *mRNA expression was lower in the HEK293 cell line than in the six RCC cell lines.

**Conclusions:**

Three putative *FABP7 *promoter regions drive reporter gene expression in RCC cell lines, but not in the HEK293 cell line. BRN2 and NFI may be key factors regulating the expression of FABP7 in certain RCC-derived cell lines.

## Background

Among primary renal tumors the most common is renal cell carcinoma (RCC). Although earlier detection of RCC has positively influenced patient outcomes [1], predicting both disease progression and patient response to treatment is difficult, due in part to the lack of suitable molecular markers [2].

FABP7 belongs to a mammalian family of at least nine proteins that are specifically expressed in diverse tissues such as liver, intestine, heart, adipose tissue, epidermis, brain, peripheral nervous system, and testis [3]. Several members of the FABP family play important roles in regulating metabolism and have been implicated in contributing to the development of insulin resistance and the metabolic syndrome [4].

Studies on human tumors and tumor-derived cell lines have indicated both FABP7's potential involvement in tumorigenesis and usefulness as a tumor marker [5-16]. Expression analyses have demonstrated *FABP7 *transcripts in tumors or urine of patients with RCC [5-8], as well as in tissues in those with glioblastoma [9] and melanoma [10,11]. *FABP7 *mRNA [5-8] and FABP7 protein [5,6,8] are overexpressed in RCC. FABP7 overexpression correlates with shorter survival in patients with glioblastoma [9,12,13] and melanoma [10,14,11], but better outcomes in those with breast cancer [15]. FABP7's role as a tumor suppressor is suggested by the finding that its enforced overexpression inhibits proliferation of a breast cancer cell line [16]. These findings clearly indicate the importance of determining how *FABP7 *expression is regulated. NFI and Pbx/POU binding sites have been found to be present in the *FABP7 *promoter in humans [17-19]. In glioma cell lines, NFI dephosphorylation is correlated with FABP7 expression [17]. In fact, all four members of the NFI family of transcription factors play key roles in the regulation of *FABP7 *in glioma cell lines [18]. Sánchez-Font et al. have suggested that *FABP7 *overexpression, controlled by the transcription factor PKNOX1, contributes to Down Syndrome-associated neurological disorders [19].

Here, we investigated the molecular mechanisms controlling FABP7 expression in human RCC cell lines.

## Results

### FABP7 expression by RCC cell lines

**Figure 1:**
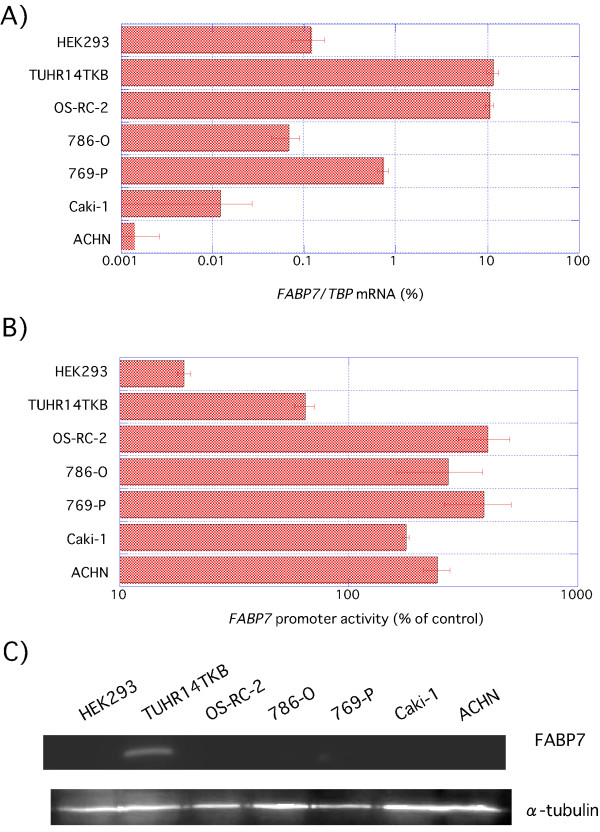
**FABP7 expression in RCC cell lines**. A) Q-PCR analysis was performed using total RNA extracted from a human embryonic kidney (HEK293) and RCC (TUHR14TKB, OS-RC-2, 786-O, 769-P, Caki-1, and ACHN) cell lines. *FABP7 *expression was determined with Q-PCR by the primers listed in Table 2. B) HEK293 and RCC (TUHR14TKB, OS-RC-2, 786-O, 769-P, Caki-1, and ACHN) cells were cotransfected with the -1122+89 pGL4-*FABP7 *promoter construct, and cultured for one day. Extracts prepared from transfected cells were assayed for luciferase activity. *FABP7 *promoter activities were normalized to the control's *Renilla *luciferase activity. The results shown are an average of four independent experiments with standard deviations indicated by the error bars. C) Western blot analysis was performed using cytoplasmic extracts from HEK293 and RCC (TUHR14TKB, OS-RC-2, 786-O, 769-P, Caki-1, and ACHN) cell lines.

We previously analyzed *FABP7 *mRNA expression in primary surgically resected RCCs and were able to detect *FABP7 *mRNA in the tumor, but not in normal tissue [6,7]. Figure 1A and Additional file 1 show the results of real-time polymerase chain reaction (quantitative PCR; Q-PCR) analysis of six RCC cell lines for *FABP7 *transcripts. Two RCC cell lines (OS-RC-2 and TUHR14TKB) exhibited strong *FABP7 *expression, in contrast to four other RCC cell lines (769-P, 786-O, ACHN and Caki-1) and a human embryonic kidney cell line (HEK293). These results differ from our analysis of primary RCCs, which expressed *FABP7 *at high frequency (80%) [7]. We also performed Western blot analysis for FABP7 expression in these same cell lines (Figure 1C, Additional file 2). A band corresponding to the major 15 kDa isoform [12] was detected at the highest level in TUHR14TKB, whereas it was not detectably expressed by HEK293 and other RCC cell lines (Figure 1C). The19 kDa FABP7 isoform potentially generated by alternative splicing (AK289836, AL512688; [20]) was not detected (Additional file 2).

### *Cis*-acting elements in the *FABP7 *promoter

**Figure 2:**
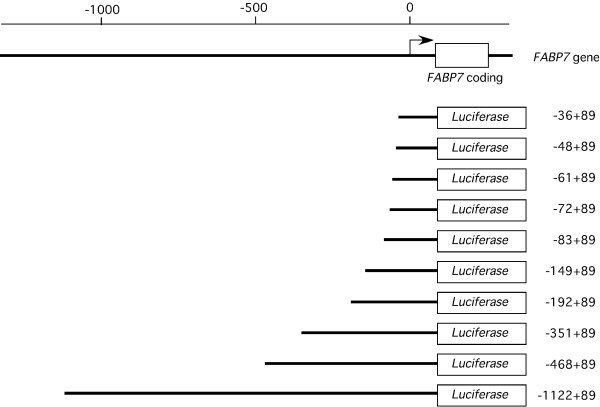
**The pGL4-*FABP7 *promoter constructs**. Schematic diagram of the *FABP7 *promoter showing the transcription start site (arrow, [17]) and coding regions (filled box). The pGL4-*FABP7 *promoter constructs extended from a common site +89 bp from the transcription start site to sites upstream (-36, -48, -61, -72, -83, -149, -192, -351, -468 and -1122 bp) were prepared by ligating PCR products to pGL4.17.

**Figure 3:**
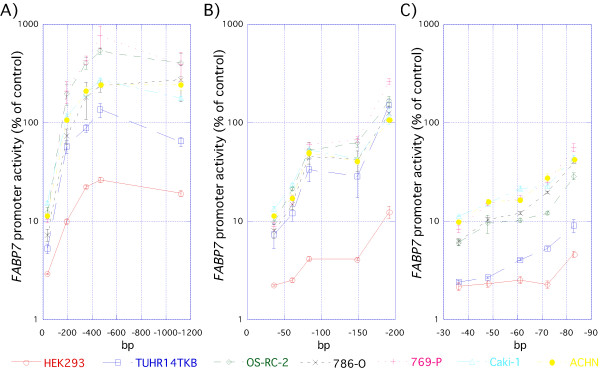
***FABP7 *promoter deletion analysis**. HEK293 and RCC (TUHR14TKB, 786-O, 769-P, OS-RC-2, Caki-1, and ACHN) cells were cotransfected with the pGL4-*FABP7 *promoter constructs described above and the pGL4.74 plasmid control, and cultured for one day. Extracts prepared from transfected cells were assayed for luciferase activity. *FABP7 *promoter activities were normalized to the control's *Renilla *luciferase activity. The results shown are an average of at least three independent experiments with standard deviations indicated by the error bars. A: Deletion analysis of the *FABP7 *promoter region. B, and C: analyses of additional deletions.

We prepared *FABP7*-luciferase constructs to determine the location of 5'-flanking *FABP7 *regulatory elements active in RCC cell lines. All promoter constructs extend from 89 base pairs (bp) downstream from the transcription start site to various upstream sites as described in Figure 2. The promoter activity of the -1122 to +89 *FABP7 *fragment in RCC cell lines was 3- to 21-fold higher than that of HEK293 (Figure 1B andAdditional file 1). *FABP7 *promoter activity did not correlate with mRNA and protein expression. The promoter deletion analyses shown in Figure 3A and Additional file 1 demonstrates the presence of a strongly positive *cis*-regulatory element between nucleotide positions -192 and -36. Interestingly, a relatively small increase (3 fold) was exhibited by the HEK293 transfectant (Figure 3A, Additional file 1). In order to more precisely localize the promoter site, we conducted detailed deletion analysis between positions -192 and -36, summarized in Figure 3B and Additional file 1. Interestingly, the positive regulatory region between -83 and -36 was more active in RCC cell lines than in HEK293, so we conducted more detail deletion analysis of the region between positions -83 and -36 (Figure 3C, Additional file 1). Compared to the promoter activity of -36+89 construct transfectant, a 2 to 3 fold increase in luciferase activity was observed with the -72+89 transfectant. In contrast, no increase was observed in HEK293 (Figure 3C, Additional file 1). The results demonstrate the presence of multiple positive *cis*-regulatory elements within the region bounded by nucleotides -72 to -36.

### Transcription factor binding to the *FABP7 *promoter

**Figure 4:**
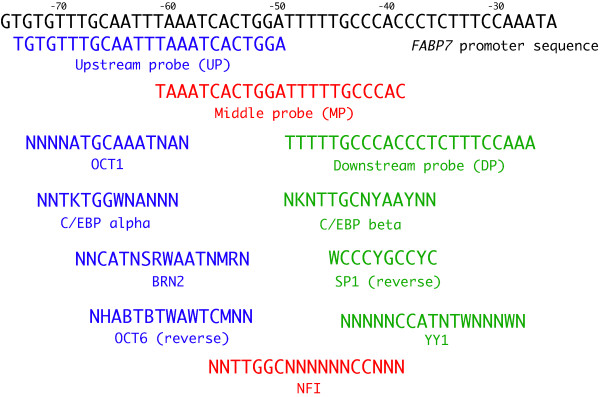
***FABP7 *promoter *cis*-regulatory region identified by promoter deletion analysis**. Numbering starts from the *FABP7 *transcription start site [17]. Oligonucleotides designated upstream (UP), middle (MP) and downstream (DP) probes are shown along with putative consensus binding sites for OCT1, C/EBP-α, BRN2, OCT6, NFI, C/EBP-β, SP1, and YY1.

We analyzed the *FABP7 *promoter region *in silico *using the TFBIND [21] and TESS [22] programs and by visual inspection with the TESS [22] to identify transcription factors binding to the *FABP7 *promoter of -72 to -36. OCT1, OCT6, BRN2, C/EBP-α, NFI, C/EBP-β, YY1, and SP1 binding sites were found across the promoter region (-73 to -28 bp) (Figure 4).

We next performed mobility shift assays to determine the factors binding to the three RCC-specific up-regulated regions of the *FABP7 *promoter. Three double-stranded oligonucleotides named upstream probe (UP), middle probe (MP), and downstream probe (DP) (Table 1 and Figure 4), which represent the active regions within the -73 to -28 sequence (-72 to -61 bp, -61 to -48 bp, and -48 to -36 bp) and used the double stranded oligonucleotides as gel shift probes.

**Table 1:** Gel shift probes

Name	Sequence Sense or Antisense
Upstream probe	TGTGTTTGCAATTTAAATCACTGGA
	TCCAGTGATTTAAATTGCAAACACA
Middle probe	TAAATCACTGGATTTTTGCCCAC
	GTGGGCAAAAATCCAGTGATTTA
Downstream probe	TTTTTGCCCACCCTCTTTCCAAA
	TTTGGAAAGAGGGTGGGCAAAAA
C/EBP alpha	TCTTTGGAAAGGT
	ACCTTTCCAAAGA
OCT1	GTAAATGCAAATCAG
	CTGATTTGCATTTAC
BRN2	GCCATTCGAAATGAGC
	GCTCATTTCGAATGGC
OCT6	GAGGAATTAGACTGC
	GCAGTCTAATTCCTC
NFI	TCTTGGCAAGAAGCCAAG
	CTTGGCTTCTTGCCAAGA
C/EBP beta	AGCTTGCACAACTC
	GAGTTGTGCAAGCT
YY1	GATAACCATTTTTGAAC
	GTTCAAAAATGGTTATC
SP1	TGAGGCAGGGT
	ACCCTGCCTCA

**Figure 5:**
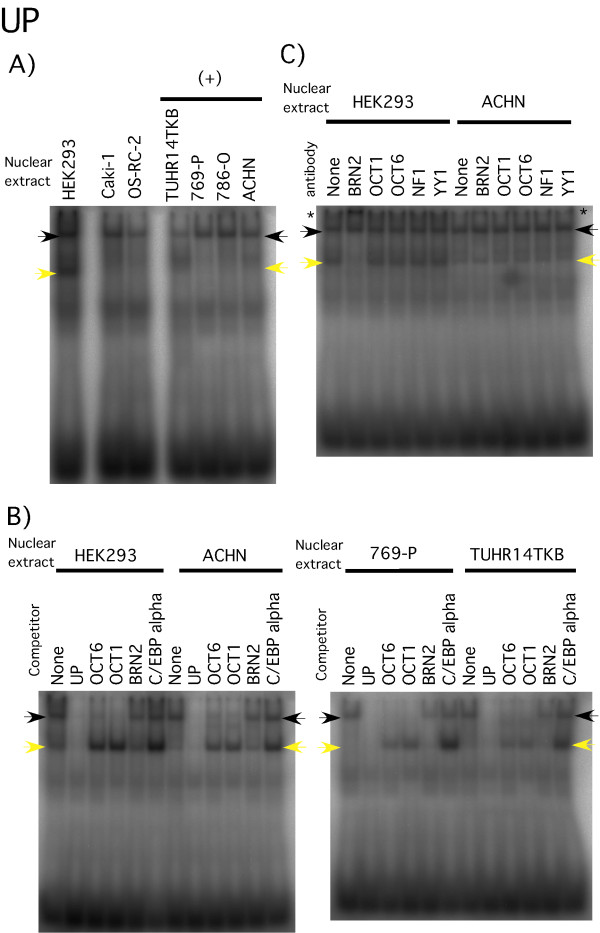
**Gel shift analysis with the UP probe**. A) ^32^P-labeled UP (Table 1 and Figure 4) were added to HEK293 or RCC (Caki-1, OS-RC-2, TUHR14TKB, 769-P, 786-O, and ACHN) nuclear extracts. In (+) cell line, luciferase activity of -72+89 vector transfectant was increased above 30% than that of -61+89 vector transfectant. Arrowheads indicate significant band shifts. B) Competition analysis. Table 1 describes the oligonucleotide sequences. A 100-fold excess of unlabeled oligonucleotide indicated in the figure was added to each reaction. C) Five micrograms of HEK293 or ACHN nuclear extracts were incubated with 2 μg of antibody (see figure) on ice for 30 min prior to addition of the ^32^P-labeled oligonucleotide probes. The '*' shows anti-BRN2 induced supershifts.

The UP detected a major band migrating at the same position in all nuclear extracts, and an additional HEK293-specific species (Figure 5A). A common shifted band (black arrows) was observed that could be competed by the addition of excess amounts of cold double-stranded OCT1 or OCT6 oligonucleotides (Figure 5B). However, the addition of anti-OCT1 or anti-OCT6 antibody to the binding reaction did not change the band shift pattern (Figure 5C). Interestingly, the addition of excess amounts of C/EBP-α, OCT1, or OCT6 probes shifted the bands up (yellow arrows) (Figure 5B). We speculated that some protein-DNA binding might prevent weak protein-DNA binding shifted up by addition of the cold probe.

**Figure 6:**
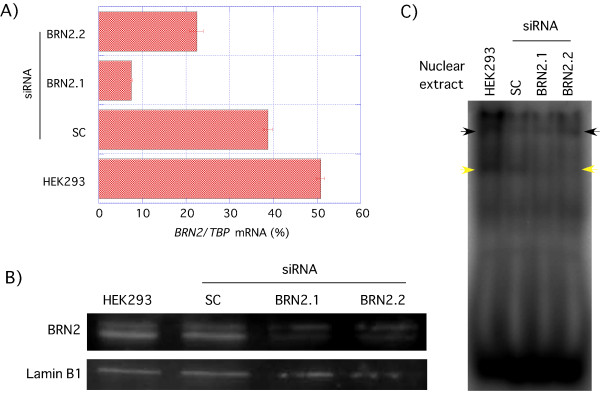
**BRN2 knockdown**. HEK293 cells were transfected with scrambled (SC) or BRN2 (BRN2.1 or BRN2.2) siRNAs. A) Q-PCR analysis for *BRN2 *to confirm efficient BRN2 knockdown. *BRN2 *was amplified using the primers shown in Table 2. B) Western blot analysis of BRN2 expression to confirm efficient BRN2 knockdown. C) Gel shift analysis for BRN2 specific band shift. ^32^P-labeled UP (Table 1 and Fig. 4) was added to nuclear extracts (5 μg) prepared from HEK293 cells transfected with scrambled (SC) or BRN2 (BRN2.1 or BRN2.2) siRNAs. Arrowheads indicate significant band shifts.

To investigate whether the HEK293-specific band (yellow arrow, Figure 5A) was BRN2-dependent, addition of BRN2 antibody to the reactions resulted in a supershift (indicated by the * in Figure 5C). We could not make clear the reason why BRN2 oligonucleotide did not change the band shift pattern. It might be the effect of band shift that shifted up the addition of excess amounts of probe (yellow arrows, Figure 5B). To demonstrate the specificity of the BRN2 antibody used for these studies, we showed that the HEK293-specific band detected by Western blot analysis was weaker when BRN2 was depleted using small interfering RNA (siRNA) (Figure 6B). Furthermore, the HEK293-specific shifted band (yellow arrow, Figure 6C) was not apparent in BRN2 specific siRNA transfectant.

**Figure 7:**
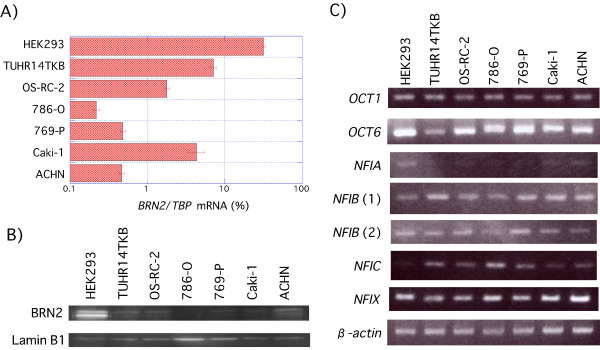
**Expression of transcription factor binding to *FABP7 *promoter**. A) Q-PCR analysis was performed using total RNA extracted from a human embryonic kidney (HEK293) and RCC (TUHR14TKB, OS-RC-2, 786-O, 769-P, Caki-1, and ACHN) cell lines. *BRN2 *was amplified using the primers shown in Table 2. B) Western blot analysis was performed using nuclear extract from a human embryonic kidney (HEK293) and RCC (TUHR14TKB, OS-RC-2, 786-O, 769-P, Caki-1, and ACHN) cell lines. C) RT-PCR analysis was performed using total RNA extracted from a human embryonic kidney (HEK293) and RCC (TUHR14TKB, OS-RC-2, 786-O, 769-P, Caki-1, and ACHN) cell lines. *OCT6*, *OCT1*, *NFIA*, *NFIB*, *NFIC*, or *NFIX *cDNAs were amplified using the primers shown in Table 2.

RCC cell lines expressed more than five times less *BRN2 *mRNA than HEK293s (Figure 7A andAdditional file 1) and BRN2 protein expression was higher in HEK293 than RCC cell lines (Figure 7B). There was an inverse correlation between *FABP7 *promoter activity (Figure 1B) and *BRN2 *mRNA expression (Figure 7A), in HEK293s and RCCs (correlation coefficient, *r *= -0.73, Additional file 1). This result indicates that BRN2 represses the *FABP7 *promoter.

**Figure 8:**
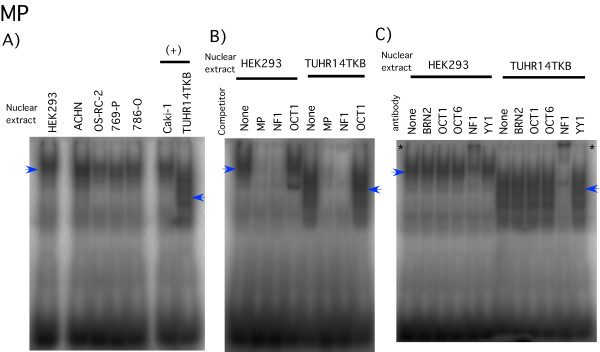
**Gel shift analysis with the MP probe**. A) ^32^P-labeled MP (Table 1 and Fig. 4) was added to HEK293 or RCC (ACHN, OS-RC-2, 769-P, 786-O, Caki-1, and TUHR14TKB) nuclear extracts. In (+) cell line, luciferase activity of -61+89 vector transfectant was increased above 30% than that of -48+89 vector transfectant. Arrowheads indicate significant band shifts. B) Competition analysis was performed using a variety of oligonucleotides (Table 1). A 100-fold excess of unlabeled competitor oligonucleotide, indicated in the figure, was added to each reaction. C) Five micrograms of HEK293 or TUHR14TKB nuclear extracts were incubated with 2 μg of the antibodies, as indicated in the figure, on ice for 30 min prior to addition of the ^32^P-labeled oligonucleotide probes. The '*' indicates anti-NFI induced supershifts.

Bisgrove et al. reported that NFI binds to the region bounded by nucleotides -54 to -40 bp of the *FABP7 *promoter [17], corresponding to the middle probe (MP), and point mutations in this region attenuate *FABP7 *promoter activity. When we performed gel shift analysis with MP, a major DNA-protein complex was observed in all seven cell lines (Figure 8A). The band marked by the blue arrow was competed by an NFI oligonucleotide in HEK293 cell line and TUHR14TKB cell line (Figure 8B). Addition of the anti-NFI antibody to the binding reaction supershifted the NFI-DNA complex in HEK293 and TUHR14TKB cell lines (indicated by the * in Figure 8C). TUHR14TKB's NFI-DNA complexes migrated differently than those of the other cell lines (Figure 8A). FABP7 expression (Figure 1C) correlated with *FABP7 *promoter activation (-61 to -48 bp) (Figure 3C and Additional file 1) and NFI gel shift patterns (Figure 8A).

The *NFI *transcription factor family includes four genes: *NFIA*, *NFIB*, *NFIC *and *NFIX *[23]. In an effort to determine which *NFI *family member or members might interact with the *FABP7 *promoter, we determined *NFI *family members' mRNA levels. *NFIA *and *NFIC *mRNA levels were, respectively, weaker and stronger in all six RCC cell lines than in HEK293 (Figure 7C). *NFIB *and *NFIX *mRNA levels were approximately the same in all seven cell lines (Figure 7C).

When DP was subjected to gel shift analysis, only one weak band was observed (Additional file 3A). This band did not compete with any of the oligonucleotides representing transcription factor binding sites, whereas addition of excess amounts of C/EBP-β probe shifted the band up (Additional file 3B). In contrast, addition of YY1 antibody did not alter this band's mobility (Additional file 3C).

## Discussion

Approximately 70% of RCC patients survive for 5 years. However, the presence of distant metastases dramatically lowers survival, indicating the importance of developing better biomarkers for predicting disease course and designing suitable therapies. Overexpression of *FABP7 *in tumor tissue and urine of patients with RCC, [5-8] led us to consider *FABP7 *as such a biomarker and to elucidate the regulation of its expression.

As an initial approach, we chose to study *FABP7 *expression in cell lines derived from human RCCs and embryonic kidney. Our finding of strong *FABP7 *mRNA expression by two of six RCC cell lines (Figure 1A) varies from our previous finding of 80% (n = 54) overexpression by carcinoma compared to corresponding expression in normal tissues [7]. Seliger et al. conducted studies related to ours that demonstrated *FABP7 *transcripts in 18 of 27 clear cell-type RCC lesions but only 4 of 19 RCC cell lines [5]. These observations may reflect the fact that higher grade (G3 + G4) versus lower grade (G1 + G2) tumors expressed significantly lower *FABP7 *mRNA levels [8]. Furthermore, ACHN and Caki-1 cell line, which expressed *FABP7 *mRNA weaker than other (Figure 1A), isolated from malignant pleural effusion and cutaneous metastasis each [24,25]. And *FABP7 *mRNA expression of 786-O cell lines was weaker than that of 769-P cell lines (Figure 1A). That RCC cases with poorer prognoses tend to express lower *FABP7 *levels, is consistent with the fact that the 786-O cell line was isolated from a patient with a prognosis less favorable than the patient from which the 769-P cell line was derived [26]. Several parallel studies have been conducted using other tumors. For example, primary versus metastatic melanomas express higher *FABP7 *mRNA levels [11]. *FABP7*-positive, compared to *FABP7*-negative, breast cancer patients experienced significantly longer disease-free survival and breast cancer specific survival [15]. The role of FABP7 as a tumor suppressor in human mammary cells is suggested by the demonstration that its enforced overexpression inhibits breast cancer cell proliferation [16]. In contrast, a recent study showed that down-regulating FABP7 using RNA interference techniques inhibited cell proliferation and migration but did not affect the invasive properties of the Caki-2 RCC cell line [27]. This suggests that the role of FABP7 in tumorigenesis depends on cell-specific regulatory factors.

We noted a discrepancy between endogenous *FABP7 *mRNA expression and the activity of the *FABP7 *promoter in cell lines derived from RCCs (786-O, ACHN, Caki-1) and that of a human embryonic kidney cell line (HEK293) (Figure 1A and 1B). These results are reminiscent of the report demonstrating that neither NFIC nor NFIX overexpression had any effect on endogenous *FABP7 *transcript levels although their overexpression decreased exogenous *FABP7 *promoter activity to 50% [18].

A possible explanation for this discrepancy is the nucleosomal organization of a chromosomal versus an episomal promoter. The basic unit of chromatin is the nucleosomal core particle, containing 147 bp of DNA that wraps twice around an octamer of core histones. The core histones bear a highly dynamic N-terminal amino acid tail approximately 20-35 residues long and rich in basic amino acids. These tails, which extend from the surface of the nucleosome, play an important role in folding of nucleosomal arrays into higher order chromatin structures that perform important functions in eukaryotic gene regulation [28]. Methylation of promoter CpG islands can play important roles in gene silencing [29]. However, this mechanism does not likely apply to human *FABP7*, since its promoter lacks a CpG island. We propose that an as-yet unidentified *cis*-acting negative regulatory element(s) residing either farther upstream or downstream from the region we analyzed in this study may explain the discrepancy between *FABP7 *mRNA levels and promoter activity.

Gene expression can be negatively regulated post-transcriptionally by microRNAs (miRNAs) [30], which play significant roles in cellular transformation and carcinogenesis by acting either as oncogenes or tumor suppressors [31]. A mechanism involving miRNAs may explain the discrepancy between *FABP7 *mRNA and protein expression levels.

Bisgrove et al. [17] reported that NFI is a key transcriptional activator of the *FABP7 *promoter in glioma cell lines. Our *FABP7 *promoter analysis reported here also showed that deletion of the NFI binding site down-regulated promoter activity in the TUHR14TKB cell line (Figure 3C), the only cell line positive for FABP7 protein expression. In glioma cell lines [17], NFI-DNA complexes were converted to a faster-migrating form by potato acid phosphatase treatment, indicating NFI activation by dephosphorylation [17]. Our gel shift analysis also revealed a similar association between the presence of a faster-migrating band (Figure 8A), *FABP7 *promoter activation (Figure 3C), and FABP7 protein expression (Figure 1C).

*NFIA *and *NFIC *were, respectively, weaker and stronger in all six RCC cell lines than in HEK293 (Figure 7C). In a glioma cell line, NFIA and NFIC siRNAs inhibited and enhanced *FABP7 *promoter activity, respectively [18], thus indicating that *FABP7 *promoter activity in RCC cell lines may be weaker than that in the HEK293 cell line. This discrepancy might be attributed to a difference in *FABP7 *promoter regulation of NFIA and NFIC in RCC cell lines and glioma cell lines.

Deletion of the mouse *Brn2 *locus results in loss of specific neuronal lineages in the hypothalamus and consequent loss of the posterior pituitary gland [32,33]. A combination of three factors, *Ascl1*, *Brn2 *and *Myt1l*, suffice to rapidly and efficiently convert mouse embryonic and postnatal fibroblasts into functional neurons *in vitro *[34]. BRN2 may coordinate normal melanocytic differentiation, whereas BRN2 can be re-activated in melanoma and may play a role in melanoma cell proliferation and tumorigenesis [35]. BRN2 also plays an important role in keratinocyte differentiation and in the pathogenesis of lichen planus lesions [36]. Our results indicate that BRN2 represses the *FABP7 *promoter and suggests that regulation of *FABP7 *expression by BRN2 differs between RCCs and melanomas.

There is a contradiction between gel shift analysis by competing with OCT1 or OCT6 probe (Figure 5B) and gel shift analysis adding anti-OCT1 or anti-OCT6 antibody (Figure 5C). One probability is that OCT1 and OCT6 probes bind to other transcription factor. However, it might be rare case for one transcription factor to bind to *FABP7 *promoter, OCT1 and OCT6 probes. Zwilling et al. reported that OCTl and OCT6 interact with high mobility group protein 2 [37]. This indicates that high mobility group protein 2 may form complex with OCT1 and OCT6. This putative complex may reflect band shift of upstream element competing with OCT1 and OCT6. Furthermore, OCT1 and OCT6 formed dimer on the consensus MORE [38]. Same complex may form on upstream element of *FABP7 *promoter. Forming OCT1-OCT6 complex might be disturbed in binding anti-OCT1 and anti-OCT6 antibodies to OCT1 and OCT6 respectively. Other possibility is that anti-OCT1 and anti-OCT6 antibodies cannot bind to OCT1 and OCT6 in our gel shift condition.

Our present finding that RCC cell lines differentially express *FABP7 *transcripts and protein, in contrast to the almost uniform overexpression of *FABP7 *transcripts by primary tumors, indicate the value of RCC cell lines for deciphering the mechanisms responsible for controlling *FABP7 *expression. Our future studies will focus on analyses of additional RCC cell lines to determine whether the low frequency of FABP7 expression is a consequence of selecting tumor cells for *in vitro *growth, and if so, will provide new insights into regulation of *FABP7 *expression. It will be important to determine whether primary tumors express FABP7 at the same high level as its transcript. If so, this would provide compelling evidence for FABP7's etiological role in RCC, and encourage future studies to evaluate it as a diagnostic marker and as a therapeutic target.

## Conclusions

A high percentage of primary RCCs, but not RCC-derived cell lines, express high *FABP7 *mRNA. The transcription factor BRN2 binds to the *cis-*acting regions in the *FABP7 *promoter identified in the present studies. BRN2 may repress the *FABP7 *promoter, because its expression inversely correlates with *FABP7 *promoter activity. NFI may also regulate FABP7 expression. Future studies will focus on analyzing these and other regulatory molecules in order to help design new therapies for treating RCC.

## Methods

### Reagents

Reagents and their sources were the following: GIBCO^® ^RPMI Media 1640, oligo (dT)_12-18_, SuperScript^® ^III Reverse Transcriptase and pCR^®^2.1-TOPO^® ^vector, Invitrogen Corp. (Carlsbad, CA); SYBR^® ^Green PCR Master Mix, Life Technologies (Carlsbad, CA); Oligopeptides and siRNAs, Hokkaido System Science (Sapporo, Hokkaido, Japan); Complete Protease Inhibitor Cocktail, FuGENE^® ^HD Transfection Reagent, poly (dI-dC) and X-tremeGENE siRNA Transfection Reagent, Roche Diagnostics GmbH (Mannheim, Germany); BCA Protein Assay Reagent Kit and NE-PER Nuclear and Cytoplasmic Extraction Reagents, Thermo Fisher Scientific (Waltham, MA); Immobilon Western HRP Substrate, Millipore (Billerica, MA); HEPES, spermidine and dithiothreitol, Sigma-Aldrich (St. Louis, MO); PVDF membrane and Illustra MicroSpin G-25 columns, GE Healthcare UK Ltd. (Little Chalfont, Buckinghamshire HP7 9NA, England); pGL4.17 and pGL4.74 plasmids and Dual-Luciferase^® ^Reporter (DLR™) Assay System, Promega (Madison, WI); restriction endonucleases and DNA Ligation Kit Ver.2.1, TAKARA BIO INC. (Otsu, Shiga, Japan); [γ-^32^P] dATP, Perkin Elmer Inc. (Waltham, MA); T4 Polynucleotide Kinase, Toyobo (Osaka, Japan); KCl, glycerol and NP-40, Wako (Osaka, Japan).

### Cell culture

RCC cell lines (786-O, 769-P and ACHN) were purchased from the American Type Culture Collection (Manassas, VA). Caki-1, HEK293, OS-RC-2, and TUHR14TKB were provided by the RIKEN (Tsukuba, Ibaraki, Japan). All cell lines were grown in GIBCO^® ^RPMI Media 1640 supplemented with 10% (v/v) fetal bovine serum (Nichirei Biosciences Inc., Japan). Cells were cultured at 37°C in a humidified atmosphere containing 5% (v/v) CO_2_.

### RT-PCR and Q-PCR analysis

Cells were cultured in 100 mm dishes for two days. Total RNA was isolated from cultured cell lines using the RNeasy Mini Kit (QIAGEN, Hilden, Germany) according to the manufacturer's instructions. Either 5 μg or 3 μg RNA was reverse transcribed using the SuperScript^® ^III Reverse Transcriptase primed by 500 ng of oligo (dT)_12-18 _according to the manufacturer's recommendations. PCR amplification was performed using 1 μL of first-strand complementary DNA (cDNA) as template with the primers and number of cycles listed in Table 2. Amplified DNA was analyzed by electrophoresis through 2% or 3% (w/v) agarose gels. Q-PCR analysis of *FABP7 *and *BRN2 *expression in RCC and HEK293 cell lines were done with the Applied Biosystems StepOnePlus™ (Life Technologies). The final PCR reaction mix included 2 μL of 5 μM specific primer, 1 μL first-strand cDNA and 10 μL SYBR^® ^Green PCR Master Mix at a final volume of 20 μL. Specific plasmid controls for FABP7, BRN2 and TBP were synthesized as the direct insertion of PCR products into a pCR^®^2.1-TOPO^® ^vector, and standard curves for each marker were generated with seven serial dilutions of plasmid templates (0.1 nM to 0.1 fM). *TBP *was used as an internal control. The sense primer for *FABP7 *amplification, primers for *TBP *amplification and primers for *OCT6 *amplification were reported by Teratani et al. [7], Jung et al. [39] and Faus et al. [40], respectively.

**Table 2:** RT-PCR and Q-PCR primers

cDNA	Sequence	Annealing temperature (°C)	Number of cycles
*FABP7 *	TGACCAACAGTCAGAACTTT	54	40
	GCCATCCCATTTCTGTATGTG		
*TBP *	TTCGGAGAGTTCTGGGATTGTA	58	40
	TGGACTGTTCTTCACTCTTGGC		
*BRN2 *	CGGCGGATCAAACTGGGATTT	57	40
	TTGCGCTGCGATCTTGTCTAT		
*OCT1 *	GCATCCAACCACCAATTTGC	57	30
	GAGGTGAGGGTGATGCTTG		
*OCT6 *	GCGGCGCATCAAGCTGGG	63	38
	CGGTTGCAGAACCAGACGCG		
*NFIA *	CAGCCAAGTGACGCTGACA	60	25
	CCTCATTGCTCCTGGACTCAT		
*NFIB *(1)	CATAACCCAGGGAACTGGAG	60	25
	CTCTTTCGTGCCATGTTCGAC		
*NFIB *(2)	CTCCCATCTGTCTCACTCAGG	58	25
	CACGGGTGCTTCTTGCCAGTC		
*NFIC *	GGACAGGGATGGGCTCTG	58	35
	CGTTCTTCTGAGGCCAGTGC		
*NFIX *	GTTTTGTGACTTCCGGGGTCTG	58	35
	GGAAGGAGGGGAGGTGATG		
*β-actin *	CAAACATGATCTGGGTCATCTTCTC	60	25
	GCTCGTCGTCGACAACGGCTC		

### Antibodies

An anti-human FABP7 Antibody (AF3166) was purchased from R&D Systems, Inc. Peroxidase-conjugated donkey anti-goat IgG (86285) was purchased from Jackson ImmunoResearch Laboratories, Inc. (West Grove, PA). A mouse monoclonal anti-α-tubulin antibody (T6074) was purchased from Sigma-Aldrich (St. Louis, MO). Goat anti-mouse IgG-HRP (sc-2005), anti-Brn-2 (C-20: sc-6029 X), anti-Lamin B (M-20: sc-6217), anti-NF-1 (H-300: sc-5567 X), anti-Oct-1 (C-21: sc-232 X), anti-Oct-6 (H-13: sc-11660 X) and anti-YY1 (H-414: sc-1703 X) were purchased from Santa Cruz Biotechnology, Inc. (Santa Cruz, CA).

### Western blot analysis

Cells were cultured in 100-mm dishes for two days. Cytoplasmic and nuclear extracts were prepared using NE-PER Nuclear and Cytoplasmic Extraction Reagents. Protein concentrations were determined using the BCA Protein Assay Reagent. For FABP7 Western blot, cytoplasmic extracts (20 μg) were electrophoresed through an 18% (w/v) polyacrylamide-SDS gel. The proteins were electrophoretically transferred onto a PVDF membrane. Membrane was incubated with 1 mg/mL FABP7 antibody diluted 1:5000, and antibody-antigen complexes were visualized with peroxidase-conjugated anti-goat IgG and Immobilon Western HRP Substrate. For BRN2 Western blot, nuclear extracts (20 μg) were electrophoresed through a 10% (w/v) polyacrylamide-SDS gel. Membrane transferring proteins was incubated with anti-Brn-2 (sc-6029 X) diluted 1:2000, and antibody-antigen complexes were visualized with peroxidase-conjugated anti-goat IgG and Immobilon Western HRP Substrate.

### Promoter constructs

The pGL4-*FABP7 *promoter construct was generated by cloning a 2.1-kb PCR fragment of the human *FABP7 *promoter region into pGL4.17. PCR reactions, using the primers listed in Table 3, were performed to generate promoter region deletions, each ending at position +89 bp from the transcription start site. Amplified DNA fragments were digested with *Bgl*II and *Hin*dIII and, using DNA Ligation Kit Ver.2.1, ligated to *Bgl*II and *Hin*dIII-digested pGL4.17 [41].

**Table 3:** PCR primers used to prepare pGL4-*FABP7 *promoter constructs

Position, cloning site and orientation	Sequence
-1122 *Bgl*II Sense	CTTTCAGATCTGGTCAGCACTAGTAAG
-468 *Bgl*II Sense	CACTAAGATCTCTCCTTTGTCTGCAAAG
-351 *Bgl*II Sense	ATCTTAGATCTTTTCCTTGCAGTCTGAG
-192 *Bgl*II Sense	GAACTAGATCTACTCCGCTAACCCAG
-149 *Bgl*II Sense	CAAAGAGATCTGGAGCCTCACTCGAGC
-83 *Bgl*II Sense	AGAGGAGATCTGGAGGGGTGTGTTTG
-72 *Bgl*II Sense	GAGAGATCTGTTTGCAATTTAAATCACTGG
-61 *Bgl*II Sense	TTTGAGATCTAAATCACTGGATTTTTGCCC
-48 *Bgl*II Sense	TCACAGATCTTTTGCCCACCCTCTTTCC
-36 *Bgl*II Sense	CCCAAGATCTTTCCAAATAAGAAGGCAG
+89 *Hin*dIII Antisense	ACAGAAAGCTTCCACCATCCTTGCCC

### Luciferase reporter assay

Human RCC and HEK293 cell lines were grown at 37°C in GIBCO^® ^RPMI Media 1640 supplemented with 10% (v/v) fetal bovine serum in a humidified, 5% CO_2 _atmosphere. Cells (5 × 10^4 ^or 7 × 10^4^) were added to each well in 24 well plates and cultured for one day. Cells were transiently co-transfected with the pGL4-*FABP7 *promoter construct (0.4 μg), pGL4.74 plasmid (0.1 μg), and 2 μL or 2.5 μL FuGENE^® ^HD Transfection Reagent. One day after transfection, luciferase activity was measured with a Dual-Luciferase^® ^Reporter (DLR™) Assay System using a Lumicounter 700 (Microtech Niti-On, Japan).

### Gel mobility shift analysis

Gel retardation assays were carried out using a slightly modified version of the method described by Bisgrove et al. [17]. Complementary oligonucleotides (Table 1) were annealed by heating at 95°C for 2 min and then cooling to room temperature. Probes were labeled with [γ-^32^P] dATP using T4 polynucleotide kinase and then purified using Illustra MicroSpin G-25 columns. Nuclear extracts were prepared using NE-PER Nuclear and Cytoplasmic Extraction Reagents. For each assay, 5 μg of nuclear extract was used. Binding reactions were performed in 30 μl binding buffer (20 mM HEPES pH 7.9, 20 mM KCl, 1 mM spermidine, 10 mM dithiothreitol, 10% (v/v) glycerol, 0.1% (v/v) NP-40, and 2 μg of poly(dI-dC)). Two microliters of 5 μM unlabeled competitor oligonucleotide or 1 μL of 2 μg/μL antibody were added as appropriate. Following addition of the nuclear extract and incubation for 30 min on ice, 1 μL of 100 nM labeled probe was added and incubated for 20 min at room temperature. DNA binding reactions were electrophoresed through a 6% (w/v) polyacrylamide gel in 0.5 × TBE to separate unbound probe from probe-protein complexes.

### siRNA transfection

The BRN-2 siRNA sequences used were as follows: 5'-GTGCAGACGCCCGTCCAG-3' (BRN2.1; [42]) and 5'-CCGCAGCGTCTAACCACTA-3' BRN2.2; [43]). The scrambled control siRNA sequence was 3'-AAGTCCATGGTGACAGGAGAC-5' (SC; [43]). The siRNA was transfected into cells using X-tremeGENE siRNA Transfection Reagent according to the manufacturer's instructions. HEK293 cells were harvested 3 to 4 days after transfection.

## List of abbreviations

cDNA: complementary DNA; FABP7: fatty acid binding protein 7; NFI: nuclear factor I; miRNA: microRNA; mRNA: messenger RNA; Q-PCR: quantitative polymerase chain reaction (real-time polymerase chain reaction); PCR: polymerase chain reaction; RCC: renal cell carcinoma; RT-PCR: reverse transcriptase-polymerase chain reaction; siRNA: small interfering RNA; SDS: sodium dodecyl sulfate; TBP: TATA box binding protein; YY1: Yin Yang 1.

## Competing interests

The authors declare that they have no competing interests.

## Authors' contributions

NT designed the study, performed the experiments, analyzed the data, and wrote the manuscript. TT1 participated in designing the study, analyzing the data and critically editing the manuscript. TT2, TS, SM and SO participated in critically editing the manuscript. All authors read and approved the final version of the manuscript.

## Supplementary Material

Additional file 1 **Data of Q-PCR and luciferase reporter assay**. Figure 1A, B, 3A, B, C and 7A - unprocessed data. Click here for file

Additional file 2 **FABP7 Western blot covering the full range**. Western blot was performed using cytoplasmic extracts from HEK293 and RCC (TUHR14TKB, OS-RC-2, 786-O, 769-P, Caki-1, and ACHN) cell lines. Click here for file

Additional file 3 **Gel shift analysis with the DP probe**. A) ^32^P-labeled DP (Table 1 and Figure 4) was added to HEK293 or RCC (769-P, 786-O, ACHN, Caki-1, OS-RC-2, and TUHR14TKB) nuclear extracts. In (+) cell line, luciferase activity of -48+89 vector transfectant was increased above 30% than that of -36+89 vector transfectant. Arrowheads indicate significant band shifts. B) Competition analysis was performed using a variety of oligonucleotides (Table 1). A 100-fold excess of unlabeled competitor oligonucleotide, indicated in the figure, was added to each reaction. C) Five micrograms of HEK293 or 786-O nuclear extracts were incubated with 2 μg of the antibodies, as indicated in the figure, on ice for 30 min prior to addition of the ^32^P-labeled oligonucleotide probes. Click here for file

## References

[B1] Weikert S Ljungberg B Contemporary epidemiology of renal cell carcinoma: perspectives of primary prevention World J Urol 2010 28 247 52 10.1007/s00345-010-0555-1 20390283

[B2] Eichelberg C Junker K Ljungberg B Moch H Diagnostic and prognostic molecular markers for renal cell carcinoma: a critical appraisal of the current state of research and clinical applicability Eur Urol 2009 55 851 863 10.1016/j.eururo.2009.01.003 19155123

[B3] Haunerland NH Spener F Fatty acid-binding proteins-insights from genetic manipulations Prog Lipid Res 2004 43 328 349 10.1016/j.plipres.2004.05.001 15234551

[B4] Boord JB Fazio S Linton MF Cytoplasmic fatty acid-binding proteins: emerging roles in metabolism and atherosclerosis Curr Opin Lipidol 2002 13 141 147 10.1097/00041433-200204000-00005 11891416

[B5] Seliger B Lichtenfels R Atkins D Bukur J Halder T Kersten M Harder A Ackermann A Malenica B Brenner W Zobawa M Lottspeich F Identification of fatty acid binding proteins as markers associated with the initiation and/or progression of renal cell carcinoma Proteomics 2005 5 2631 2640 10.1002/pmic.200401264 15892167

[B6] Domoto T Miyama Y Suzuki H Teratani T Arai K Sugiyama T Takayama T Mugiya S Ozono S Nozawa R Evaluation of S100A10, annexin II and B-FABP expression as markers for renal cell carcinoma Cancer Sci 2007 98 77 82 10.1111/j.1349-7006.2006.00355.x 17083565 PMC11159138

[B7] Teratani T Domoto T Kuriki K Kageyama T Takayama T Ishikawa A Ozono S Nozawa R Detection of transcript for brain-type fatty Acid-binding protein in tumor and urine of patients with renal cell carcinoma Urology 2007 69 236 240 10.1016/j.urology.2006.09.060 17320655

[B8] Tölle A Jung M Lein M Johannsen M Miller K Moch H Jung K Kristiansen G Brain-type and liver-type fatty acid-binding proteins: new tumor markers for renal cancer? BMC Cancer 2009 9 248 10.1186/1471-2407-9-248 19622156 PMC2732640

[B9] Liang Y Diehn M Watson N Bollen AW Aldape KD Nicholas MK Lamborn KR Berger MS Botstein D Brown PO Israel MA Gene expression profiling reveals molecularly and clinically distinct subtypes of glioblastoma multiforme Proc Natl Acad Sci USA 2005 102 5814 5819 10.1073/pnas.0402870102 15827123 PMC556127

[B10] Goto Y Matsuzaki Y Kurihara S Shimizu A Okada T Yamamoto K Murata H Takata M Aburatani H Hoon DS Saida T Kawakami Y A new melanoma antigen fatty acid-binding protein 7, involved in proliferation and invasion, is a potential target for immunotherapy and molecular target therapy Cancer Res 2006 66 4443 4449 10.1158/0008-5472.CAN-05-2505 16618771

[B11] Goto Y Koyanagi K Narita N Kawakami Y Takata M Uchiyama A Nguyen L Nguyen T Ye X Morton DL Hoon DS Aberrant fatty acid-binding protein-7 gene expression in cutaneous malignant melanoma J Invest Dermatol 2010 130 221 229 10.1038/jid.2009.195 19587692 PMC2845961

[B12] Liang Y Bollen AW Aldape KD Gupta N Nuclear FABP7 immunoreactivity is preferentially expressed in infiltrative glioma and is associated with poor prognosis in EGFR-overexpressing glioblastoma BMC Cancer 2006 6 97 10.1186/1471-2407-6-97 16623952 PMC1479358

[B13] Kaloshi G Mokhtari K Carpentier C Taillibert S Lejeune J Marie Y Delattre JY Godbout R Sanson M FABP7 expression in glioblastomas: relation to prognosis, invasion and EGFR status J Neurooncol 2007 84 245 248 10.1007/s11060-007-9377-4 17415524

[B14] Slipicevic A Jørgensen K Skrede M Rosnes AK Trøen G Davidson B Flørenes VA The fatty acid binding protein 7 (FABP7) is involved in proliferation and invasion of melanoma cells BMC Cancer 2008 8 276 10.1186/1471-2407-8-276 18826602 PMC2569959

[B15] Zhang H Rakha EA Ball GR Spiteri I Aleskandarany M Paish EC Powe DG Macmillan RD Caldas C Ellis IO Green AR The proteins FABP7 and OATP2 are associated with the basal phenotype and patient outcome in human breast cancer Breast Cancer Res Treat 2010 121 41 51 10.1007/s10549-009-0450-x 19590950

[B16] Shi YE Ni J Xiao G Liu YE Fuchs A Yu G Su J Cosgrove JM Xing L Zhang M Li J Aggarwal BB Meager A Gentz R Antitumor activity of the novel human breast cancer growth inhibitor, mammary-derived growth inhibitor-related gene, *MRG* Cancer Res 1997 57 3084 3091 9242429

[B17] Bisgrove DA Monckton EA Packer M Godbout R Regulation of Brain Fatty Acid-binding Protein Expression by Differential Phosphorylation of Nuclear Factor I in Malignant Glioma Cell Lines J Biol Chem 2000 275 30668 30676 10896661 10.1074/jbc.M003828200

[B18] Brun M Coles JE Monckton EA Glubrecht DD Bisgrove D Godbout R Nuclear factor I regulates brain fatty acid-binding protein and glial fibrillary acidic protein gene expression in malignant glioma cell lines J Mol Biol 2009 391 282 300 10.1016/j.jmb.2009.06.041 19540848

[B19] Sánchez-Font MF Bosch-Comas A Gonzàlez-Duarte R Marfany G Overexpression of *FABP7 *in Down syndrome fetal brains is associated with *PKNOX1 *gene-dosage imbalance Nucleic Acids Res 2003 31 2769 2777 10.1093/nar/gkg396 12771203 PMC156729

[B20] NCBI FABP7 gene resource http://www.ncbi.nlm.nih.gov/gene/2173

[B21] Tsunoda T Takagi T Estimating transcription factor bindability on DNA Bioinformatics 1999 15 622 630 10.1093/bioinformatics/15.7.622 10487870

[B22] Transcription Element Search System http://www.cbil.upenn.edu/cgi-bin/tess/tess

[B23] Gronostajski RM Roles of the NFI/CTF gene family in transcription and development Gene 2000 249 31 45 10.1016/S0378-1119(00)00140-2 10831836

[B24] Kochevar J A renal cell carcinoma neoplastic antigen detectable by immunohistochemistry is defined by a murine monoclonal antibody Cancer 1987 60 2031 2036 10.1002/1097-0142(19870615)59:12<2031::aid-cncr2820591211>3.0.co;2-0 3552200

[B25] Fogh J Trempe G Fogh J New human tumor cell lines Human Tumor Cells In Vitro 1975 New York: Plenum Press 115 141

[B26] Williams RD Elliott AY Stein N Fraley EE In vitro cultivation of human renal cell cancer. II. Characterization of cell lines In Vitro 1978 14 779 786 10.1007/BF02617972 721102

[B27] Tölle A Krause H Miller K Jung K Stephan C Importance of brain-type fatty acid binding protein for cell-biological processes in human renal carcinoma cells Oncol Rep 2011 25 1307 1312 21399875 10.3892/or.2011.1209

[B28] Munshi A Shafi G Aliya N Jyothy A Histone modifications dictate specific biological readouts J Genet Genomics 2009 36 75 88 10.1016/S1673-8527(08)60094-6 19232306

[B29] Takai D Jones PA Comprehensive analysis of CpG islands in human chromosomes 21 and 22 Proc Natl Acad Sci USA 2002 99 3740 3745 10.1073/pnas.052410099 11891299 PMC122594

[B30] Bartel DP MicroRNAs: target recognition and regulatory functions Cell 2009 136 215 233 10.1016/j.cell.2009.01.002 19167326 PMC3794896

[B31] Wiemer EA The role of microRNAs in cancer: no small matter Eur J Cancer 2007 43 1529 1544 10.1016/j.ejca.2007.04.002 17531469

[B32] Nakai S Kawano H Yudate T Nishi M Kuno J Nagata A Jishage K Hamada H Fujii H Kawamura K Shiba K Noda T The POU domain transcription factor Brn-2 is required for the determination of specific neuronal lineages in the hypothalamus of the mouse Genes Dev 1995 9 3109 3121 10.1101/gad.9.24.3109 8543155

[B33] Schonemann MD Ryan AK McEvilly RJ O'Connell SM Arias CA Kalla KA Li P Sawchenko PE Rosenfeld MG Development and survival of the endocrine hypothalamus and posterior pituitary gland requires the neuronal POU domain factor Brn-2 Genes Dev 1995 9 3122 3135 10.1101/gad.9.24.3122 8543156

[B34] Vierbuchen T Ostermeier A Pang ZP Kokubu Y Südhof TC Wernig M Direct conversion of fibroblasts to functional neurons by defined factors Nature 2010 463 1035 1041 10.1038/nature08797 20107439 PMC2829121

[B35] Cook AL Sturm RA POU domain transcription factors: BRN2 as a regulator of melanocytic growth and tumourigenesis Pigment Cell Melanoma Res 2008 21 611 626 10.1111/j.1755-148X.2008.00510.x 18983536

[B36] Shi G Sohn KC Choi DK Kim YJ Kim SJ Ou BS Piao YJ Lee YH Yoon TJ Lee Y Seo YJ Kim CD Lee JH Brn2 is a transcription factor regulating keratinocyte differentiation with a possible role in the pathogenesis of lichen planus PLoS One 2010 5 e13216 10.1371/journal.pone.0013216 20967260 PMC2953493

[B37] Zwilling S König H Wirth T High mobility group protein 2 functionally interacts with the POU domains of octamer transcription factors EMBO J 1995 14 1198 1208 7720710 10.1002/j.1460-2075.1995.tb07103.x PMC398197

[B38] Tomilin A Reményi A Lins K Bak H Leidel S Vriend G Wilmanns M Schöler HR Synergism with the coactivator OBF-1 (OCA-B, BOB-1) is mediated by a specific POU dimer configuration Cell 2000 103 853 864 10.1016/S0092-8674(00)00189-6 11136971

[B39] Jung M Ramankulov A Roigas J Johannsen M Ringsdorf M Kristiansen G Jung K In search of suitable reference genes for gene expression studies of human renal cell carcinoma by real-time PCR BMC Mol Biol 2007 8 47 10.1186/1471-2199-8-47 17559644 PMC1913536

[B40] Faus I Hsu HJ Fuchs E Oct-6: a regulator of keratinocyte gene expression in stratified squamous epithelia Mol Cell Biol 1994 14 3263 3275 7909356 10.1128/mcb.14.5.3263-3275.1994 PMC358693

[B41] Takaoka N Fukuzawa M Saito T Sakaitani T Ochiai H Promoter analysis of the membrane protein gp64 gene of the cellular slime mold *Polysphondylium pallidum* Biochim Biophys Acta 1999 1447 226 230 10542319 10.1016/s0167-4781(99)00179-7

[B42] Goodall J Carreira S Denat L Kobi D Davidson I Nuciforo P Sturm RA Larue L Goding CR Brn-2 represses microphthalmia-associated transcription factor expression and marks a distinct subpopulation of microphthalmia-associated transcription factor-negative melanoma cells Cancer Res 2008 68 7788 7794 10.1158/0008-5472.CAN-08-1053 18829533

[B43] Wellbrock C Rana S Paterson H Pickersgill H Brummelkamp T Marais R Oncogenic BRAF regulates melanoma proliferation through the lineage specific factor MITF PLoS One 2008 3 e2734 10.1371/journal.pone.0002734 18628967 PMC2444043

