# Supplementary figures and images for: Analysis of the regulation of fatty acid binding protein 7 expression in human renal carcinoma cell lines
Source: BMC Mol Biol. 2011 Jul 19;12:31. doi: 10.1186/1471-2199-12-31 (PMC3162894; doi:10.1186/1471-2199-12-31)

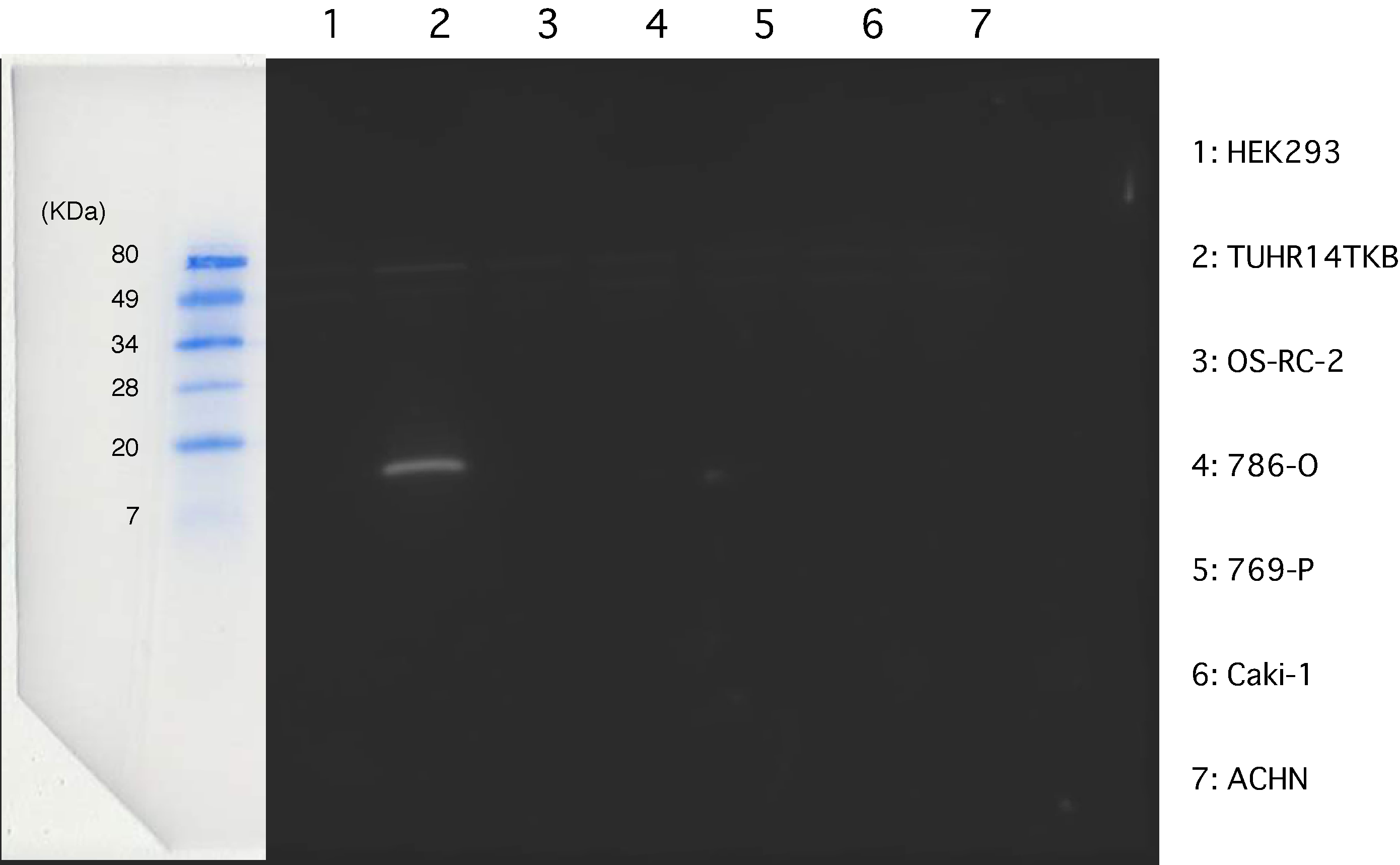

Supplement: Additional file 2 — FABP7 Western blot covering the full range. Western blot was performed using cytoplasmic extracts from HEK293 and RCC (TUHR14TKB, OS-RC-2, 786-O, 769-P, Caki-1, and ACHN) cell lines. [file 1471-2199-12-31-S2.BMP]

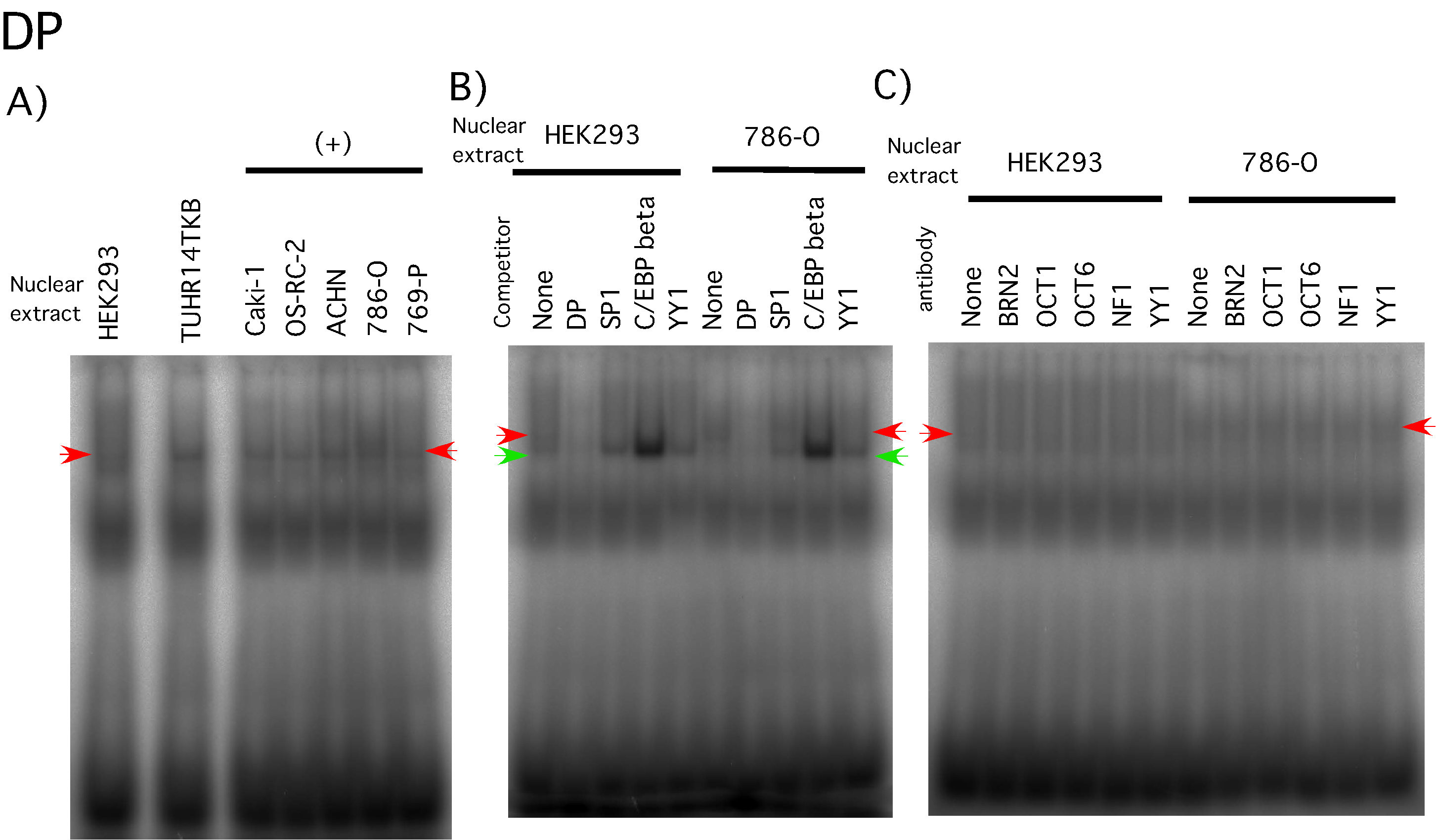

Supplement: Additional file 3 — Gel shift analysis with the DP probe. A) 32P-labeled DP (Table 1 and Figure 4) was added to HEK293 or RCC (769-P, 786-O, ACHN, Caki-1, OS-RC-2, and TUHR14TKB) nuclear extracts. In (+) cell line, luciferase activity of -48+89 vector transfectant was increased above 30% than that of -36+89 vector transfectant. Arrowheads indicate significant band shifts. B) Competition analysis was performed using a variety of oligonucleotides (Table 1). A 100-fold excess of unlabeled competitor oligonucleotide, indicated in the figure, was added to each reaction. C) Five micrograms of HEK293 or 786-O nuclear extracts were incubated with 2 μg of the antibodies, as indicated in the figure, on ice for 30 min prior to addition of the 32P-labeled oligonucleotide probes. [file 1471-2199-12-31-S3.BMP]
